# Supplementary material for: Chromothripsis during telomere crisis is independent of NHEJ, and consistent with a replicative origin
Source: Genome Res. 2019 May;29(5):737–49. doi: 10.1101/gr.240705.118 (PMC6499312; doi:10.1101/gr.240705.118)
Supplement: Supplemental Material [file supp_gr.240705.118_Supplemental_file_1.zip › contigs/annotated_contigs/DB116/contig.2.DB116_length_332_mean_cov_6.04819277108.docx]

**DB116_length_332_mean_cov_6.04819277108**

```
TCTCTGGTAGACATGGTGGAGTGGTAAGTGCACCGACATTTCTCACATCCTGGAGGAAGCTGTGAACATTCTGAGGACTACTGCCGGCT
 >chr9:138348189-138348475 - E=4e-152 p=0e+00
TCAGTGCCCCTGTTGGGATTTTCATGGATGATCCTGGGGAGGGCGGCTTCGCCTTTCTGAAACACCTTCTCCTGGTGGAGGTGGCCCAA

TCCCCCACCCTGTAACTCCATCTCTCCCCTCATCTCTCCCCTCCCCTCCATCCCTCCCCGACCTCCATCCCTCCTCTCACCTCCATCCC

TCCCCTCACCTCCCTCCCT|TCCCCTCCCCTCCCTCCCTTCCCCTCCCCTCCCTCCCTCCCCTC|AC                     >chr8:109988350-109988397 + E=1e-09
```
